# Investigating the human jejunal microbiota

**DOI:** 10.1038/s41598-022-05723-9

**Published:** 2022-01-31

**Authors:** Heidi Cecilie Villmones, Marius Svanevik, Elling Ulvestad, Tore Stenstad, Inger Lill Anthonisen, Randi Monsen Nygaard, Ruben Dyrhovden, Øyvind Kommedal

**Affiliations:** 1 Department of Microbiology, Vestfold Hospital Trust, Postbox 2168, 3103 Tønsberg, Norway; 2 Department of Gastrointestinal Surgery, Vestfold Hospital Trust, 3103 Tønsberg, Norway; 3 Morbid Obesity Center, Vestfold Hospital Trust, 3103 Tønsberg, Norway; 4 Department of Clinical Science, University of Bergen, 5021 Bergen, Norway; 5 Department of Infectious Medicine, Vestfold Hospital Trust, 3103 Tønsberg, Norway; 6 Department of Microbiology, Haukeland University Hospital, 5021 Bergen, Norway

**Keywords:** Microbiology, Molecular biology, Anatomy, Gastroenterology, Medical research, Molecular medicine

## Abstract

Descriptions of the small intestinal microbiota are deficient and conflicting. We aimed to get a reliable description of the jejunal bacterial microbiota by investigating samples from two separate jejunal segments collected from the luminal mucosa during surgery. Sixty patients with morbid obesity selected for elective gastric bypass surgery were included in this survey. Samples collected by rubbing a swab against the mucosa of proximal and mid jejunal segments were characterized both quantitatively and qualitatively using a combination of microbial culture, a universal quantitative PCR and 16S deep sequencing. Within the inherent limitations of partial 16S sequencing, bacteria were assigned to the species level. By microbial culture, 53 patients (88.3%) had an estimated bacterial density of < 1600 cfu/ml in both segments whereof 31 (51.7%) were culture negative in both segments corresponding to a bacterial density below 160 cfu/ml. By quantitative PCR, 46 patients (76.7%) had less than 10^4^ bacterial genomes/ml in both segments. The most abundant and frequently identified species by 16S deep sequencing were associated with the oral cavity, most often from the *Streptococcus mitis group,* the *Streptococcus sanguinis group, Granulicatella adiacens/para-adiacens,* the *Schaalia odontolytica complex* and *Gemella haemolysans/taiwanensis*. In general, few bacterial species were identified per sample and there was a low consistency both between the two investigated segments in each patient and between patients. The jejunal mucosa of fasting obese patients contains relatively few microorganisms and a core microbiota could not be established. The identified microbes are likely representatives of a transient microbiota and there is a high degree of overlap between the most frequently identified species in the jejunum and the recently described ileum core microbiota.

## Introduction

The longstanding debate as to whether antibacterial mechanisms of the intestinal epithelium along with peristalsis prevent the formation of a resident jejunal microbiota, is still not resolved^1–4^. Descriptions of the jejunal microbiota remains vague and there is little consistency both among microbial quantifications and described microbial compositions. Contemporary textbooks^5^ and reviews^6–12^ report bacterial concentrations of 10^4^ to 10^7^ cfu/ml, dominated by lactobacilli, streptococci, enterococci and *Veillonella* spp. *Enterobacteriales* are also considered to be prominent participants^8–10,13,14^.

Deep sequencing approaches^13–21^ have failed to define a consistent core microbiota. *Streptococcus*, *Prevotella, Veillonella* and *Fusobacterium* are frequently detected genera along with a range of Proteobacteria including *Enterobacteriales, Haemophilus spp*. and *Neisseria spp*. These studies are typically based on indirect sample collection procedures like endoscopies^13,14,16,17,19,20,22^, nasoileal catheters^18,23^, capsules^15^ or from autopsies^21^. Despite the use of indirect sampling, the possibility for sample contamination from more abundantly colonized parts of the gastrointestinal tract has rarely been addressed.

We have identified four older studies on samples collected directly from the jejunal lumen during surgery^3,24–26^. These studies, published between 1953 and 1979 were based on culture-dependent techniques. They consistently report a high proportion of jejunal samples to be sterile, 71%, 20%, 63% and 69% respectively. The sporadic species detected, typically gram-positive facultative bacteria like viridans streptococci, were related to the oral cavity and generally considered transient microorganisms. Strict anaerobes, *Enterobacteriales* and enterococci were rarely detected.

In an attempt to provide a comprehensive and methodically sound description of the jejunal microbiota, we collected samples from two separate jejunal segments in a cohort of 60 patients during scheduled gastric bypass surgery. The samples were characterized qualitatively and quantitatively using a combination of microbial culture, a universal quantitative PCR and 16S deep sequencing. The study population was a selected group of patients with morbid obesity otherwise considered intestinally healthy. Although some components of their microbiota might differ from that of a normal weight population, we believe the overall findings will be representative and can contribute to our understanding of the normal human jejunal microbiota.

## Results

### Patient characteristics

A total of 60 patients were included with a median age of 45 years and a preponderance of females (70%). All patients were intestinally healthy, but due to morbid obesity and other comorbid conditions most are classified with an ASA risk score 3 (Table 1).

**Table 1:** Patient characteristics.

Population characteristics	Number of patients (n = 60)	Percent
Age years, median (range)	45 (19–65)	
BMI kg/m^2^, median (range)	41 (34–57)	
Gender, male	18	30
Gender, female	42	70
ASA score 1	0	0
ASA score 2	3	5
ASA score 3	56	93
ASA score 4	1	2
Current smoker	0	0
Systolic BP, mean (95%CI)	136 (132–140)	
Diastolic BP, mean (95%CI)	85 (83–87)	
**Comorbidities**	49	82
Diabetes, any type	10	17
Peroral antidiabetics	7	12
Insulin dependant	1	2
Hypertension	21	35
Dyslipidemia	12	20
Obstructive sleep apnea (OSA)	17	28
OSA with CPAP	15	25
Previous cholecystectomy	10	17
Proton pump inhibitor	14	23

	Median (range)	
Operative time, min	56 (31–101)	
Postoperative stay, days	1 (1–7)	

*BMI* Body Mass Index, *ASA* American Society of Anesthesiologists, *BP* blood pressure, *CPAP* continuous positive airway pressure.

### Findings by microbial culture

Bacterial concentrations as estimated by microbial culture are presented in Table 2. No growth in either segment was observed for 31 patients (51.7%) and only three patients (5%) had growth that exceeded the upper limit of quantification (> 1.6 × 10^4^). Cultivated bacteria are listed in Supplementary Table S1. When combining results from both jejunal segments, the most frequent bacteria at the patient level were: *Streptococcus salivarius/vestibularis* (25% of patients), *S. parasanguinis* (16%), *S. mitis/oralis* (12%), *Rothia mucilaginosa* (10%), *Actinomyces odontolyticus (8%), Haemophilus parainfluenzae (8%), Neisseria flavescens/subflava (5%)* and *Neisseria parahaemolyticus (5%). Enterobacteriales* were only detected in one patient (a *Klebsiella pneumoniae*). Fungi, a *Candida albicans,* grew in only one sample collected from a mid-segment.

**Table 2:** Microbiological densities estimated in jejunum by aerobic and anaerobic culture.

Growth	cfu/ml	Proximal jejunum (*n*)	Mid jejunum (*n*)	Patient level (*n*) (Both segments combined)*	
No growth	< 160	34	43	51.7% (31)	88.3%
Single colony/broth only	160– < 1600	21	13	36.7% (22)	
Sparse growth	1600– < 8000	3	0	3.3% (2)	11.6%
Moderate growth	0.8–1.6 × 10^4^	1	2	3.3% (2)	
Abundant growth	> 1.6 × 10^4^	1	2	5% (3)	
In total		60	60	100%	100%

*Counted by the most bacteria rich segment.

### Deep sequencing technical results

The median number of reads per sample was 445,263 (range 264,689 to 911,244). After removal of chimera, small OTUs (< 50) and contaminants, the median number of remaining reads was 19,568 (range 2700 to 282,249). The median percentage of retained reads was 9.9% (range 0.5% to 97.4%) with only 16 of 120 samples having more than half of the reads left.

### Microbial findings by 16S deep sequencing

**Figure 1:**
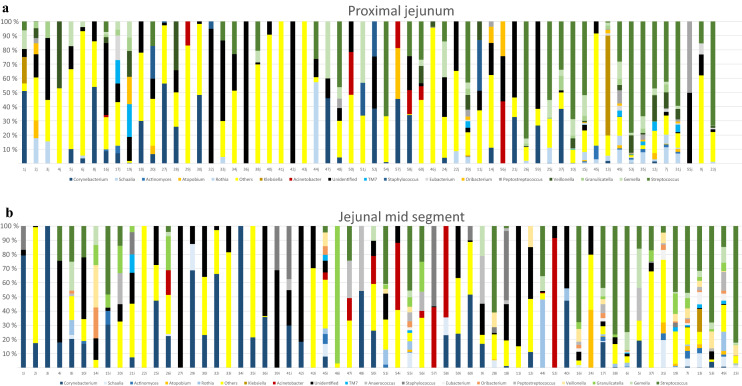
Relative distribution of most abundant genera in (**a**) proximal part of jejunum and (**b**) jejunal mid-segment. All 60 patients included. Samples are sorted by increasing bacterial concentration. Samples with concentration below the level of quantification (Ct-value ≥ 34.17) are sorted by name on the left side (35 proximal samples 1j-60j and 38 mid-segment samples, 1i-60i).

In total, after filtering of possible contaminants, we identified 218 different species (Supplementary Table S2). A per sample description at the genus level is provided in Fig. 1 and at the phylum level in Supplementary Fig. S1. Actinobacteria, especially *Corynebacterium* *spp*., seems to be more abundant in samples with low bacterial loads whereas the presence of Firmicutes, in particular *Streptococcus* *spp*., *Gemella* *spp*. and *Granulicatella* *spp*. increases in samples with higher bacterial loads.

**Figure 2:**
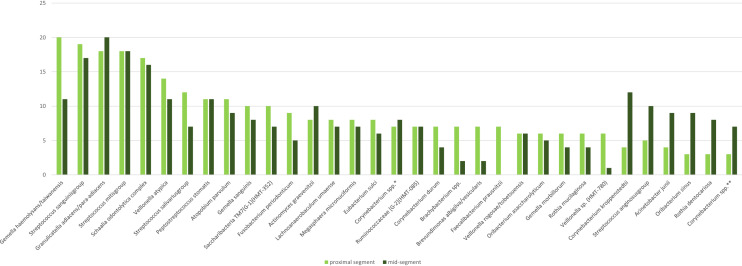
Comparison of most frequent species in upper and middle jejunum. (y axis number of patients). Based on all 60 patients.

An overall species-level comparison of results from the upper and middle part of jejunum revealed no noticeable differences (Fig. 2). In addition, most species were found at low concentrations close to the limit of detection and consequently prone to random detection. Therefore, for the remaining part of this manuscript, results from the two segments were merged and reported per patient. No species was detected in more than 50% of the population and only six species/groups of species were found in more than 30% of participants: *Enterobacteriales* were only exceptionally detected; *Escherichia coli* in 3 patients (5%), *Serratia grimesii/proteamaculans/liquefaciens* in 3 patients (5%) and *Klebsiella pneumonia complex* in 2 patients (3%).

### Quantification by PCR

Bacterial genome concentrations as determined by the quantitative 16S rRNA PCR are presented in Table 3. The median density was found to be below our limit of genomic quantification i.e. < 2.9 × 10^3^ bacterial genomes per ml.

**Table 3:** Microbial densities estimated by quantitative 16S rRNA PCR.

Genome copies/ml	Proximal jejunum (*n*)	Mid jejunum (*n*)	Patient level (n) (Both segments combined)*
10^5^– < 10^6^	5% (3)	5% (3)	5% (3)
10^4^– < 10^5^	16.7% (10)	6.7% (4)	18.3% (11)
2.9 × 10^3^– < 10^4^	20% (12)	25% (15)	36.7% (22)
< 2.9 × 10^3^	58.3% (35)	63.3% (38)	40% (24)

*Counted by the most microbial genome-rich segment.

Intra-patient concentration differences between proximal and mid-jejunal samples were generally small, and there was no overall tendency towards a higher bacterial load in neither of the segments. In four patients (9, 49, 53 and 55) we observed a more than tenfold concentration difference between the two samples, two of them with the highest load in the proximal sample and two with the highest load in the more distal sample.

### Results from microbial culture and 16S deep sequencing compared

A comparison of findings by microbial culture versus by deep sequencing is provided in Supplementary Table S3. As expected, deep sequencing identified far more species than culture. Out of 120 samples, only 43 were culture positive. Still, culture made 22 identifications not reproduced by sequencing. These identifications were most often from the species *Haemophilus parainfluenzae, Actinomyces odontolyticus, Micrococcus luteus, Streptococcus mitis group* and *Streptococcus salivarius/vestibularis.*

### Comparison of sequencing results from this study with previously reported results from the ileum

**Figure 3:**
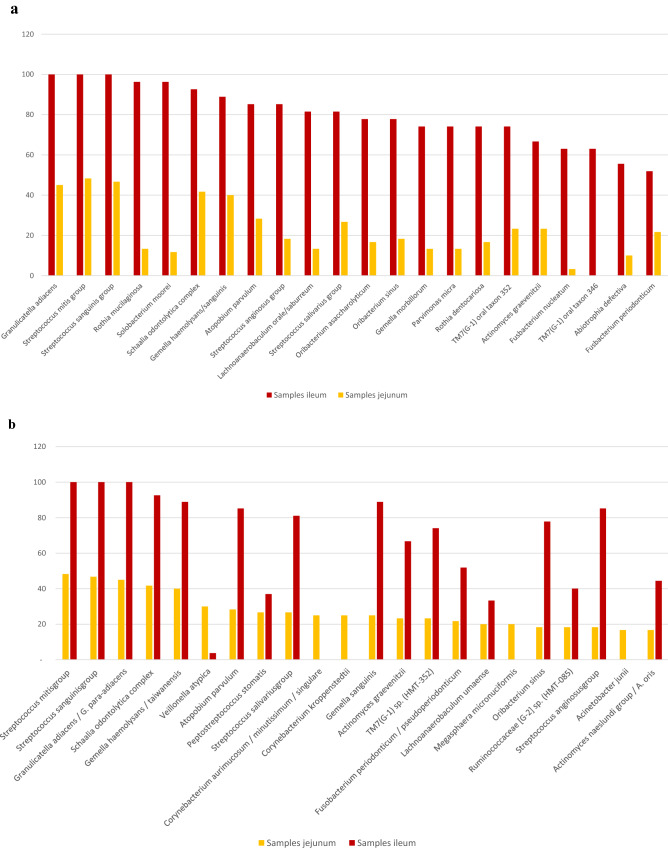
(**a**) Previously reported ileum core microbiota (based on 27 patients) sorted by frequency (%) compared to observed frequency in jejunum (upper and middle segment combined for all 60 patients) and (**b**). Most frequently detected species in jejunum (upper and middle segment combined) sorted by frequency (%) compared to previously reported frequency in ileum. Based on all 60 patients.

In a recent study on peroperative ileal samples from patients undergoing radical cystectomy^27^ we defined an ileum core microbiota consisting of 22 species, each present in more than half of the patients. In Fig. 3a, we show that except from the provisional species *TM7(G-1) oral taxon 346,* all 22 ileum core microbiota species were also detected in the jejunal samples although much more sporadically. In Fig. 3b we compare the most frequent species in jejunum with their corresponding frequencies in ileum. Except from *Corynebacterium kroppenstedtii, C. aurimucosum/minutissimum/singulare* and *Acinetobacter junii*, all these were also part of the ileum core microbiota. Again, most of them were much more frequently detected in ileum. *Granulicatella adiacens, Streptococcus mitis group* and *Streptococcus sanguinis group* were the three most frequent identifications in both ileum and jejunum.

## Discussion

Our results indicate that the jejunum is more scarcely populated by bacteria than specified by contemporary reviews and textbooks. Using a combination of cultivation and deep sequencing with a limit of detection in the range 10^2^ to 10^3^ cfu/genomes per milliliter of sample material, we were unable to recover a resident core microbiota in the proximal and middle parts of the human jejunum.

Keystone species are thought to be important for shaping the organization and diversity of an ecological community, and a common core microbiota has been variably defined as microbial taxa present in between 30 and 95% of a population^28^. In our material, only five identifications were made in more than 30% of the patients: *Streptococcus mitis group* (48%), *Streptococcus sanguinis group* (47%), *Granulicatella adiacens/para-adiacens* (45%), *Schaalia odontolytica complex* (42%) and *Gemella haemolysans/taiwanensis* (40%). All of these are groups of species not possible to differentiate by 16S rRNA sequencing, and therefore most likely, as previously demonstrated in ileum^27^, represent more than one species each. Further, microbial resemblance between samples was low both between the upper and middle segments in each patient and between patients.

We detected a wide range of *Corynebacterium* species in this study, and they also dominated in some of the weak positive samples. Among these, *Corynebacterium kroppenstedtii*, *Corynebacterium aurimucosum* and *Corynebacterium durum* were detected rather frequently (18–25% of patients). *Corynebacterium kroppenstedtii* was first isolated from a sputum sample, *C. durum* from respiratory samples and *C. aurimucosum* from human clinical samples. Also the other Corynebacteria deemed relevant in this study are associated with the human microbiota and/or human infections with the exception of *C. vitaeruminis*, a vitamin-B producing microbe isolated from the rumen of cows. It could be argued that *C. vitaeruminis* should be removed as biologically unexpected, but is was detected in seven patient samples.

The jejunal samples also generally revealed low bacterial densities, both by cultivation and by 16S rRNA quantification. The median density was < 1600 cfu/ml or < 2.9 × 10^3^ bacterial genomes/ml. Only 23.3% of the samples had an estimated bacterial concentration above 10^4^ genome copies per ml. In contrast to cultivation, 16S deep sequencing was positive in all samples, although sometimes only with a single species.

Despite being performed on two different patient populations, the overall spectrum of bacteria identified from the jejunal samples in the present study, bears a striking resemblance to the ileal core microbiota as defined by our recent studies on surgically collected ileal samples (Fig. 3a,b)^27,29^. 16S Ct-values indicate that the bacterial concentration in ileum is at least 100 fold higher than in jejunum. The overall impression is that bacterial cells are capable of multiplying and forming a stable microbiota with a definable core microbiota only more distally in the ileum. Consequently, our results revitalize discussions as to whether the human jejunum harbors only transient microbes and no resident microbiota.

The higher bacterial concentrations in jejunum reported by previous 16S deep sequencing studies might reflect the use of indirect sample collection methods by endoscopies, nasoileal catheters and capsules with an inherent risk of contamination from the more abundantly colonized upper gastrointestinal tract. The human oral cavity has a rich and dense microbiota^30^ and saliva is estimated to contain 10^9^ cfu/ml^31^. Provided a bacterial density of maximum 10^3^–10^4^ in jejunum, this represents a gradient of at least 1:10^5^ suggesting that both endoscopies and nasoileal tubes introduce a considerable risk for contamination^32^. This can also affect microbial composition. In a study using endoscopy to collect samples^33^ they found *Bacteroides, Prevotella* and *Helicobacter* to be among the most frequent and dominant genera. Although we also found multiple species from these genera in our samples they were mainly represented as only minor constituents. To the best of our knowledge, the present study is the first to combine 16S deep sequencing with samples collected directly from the jejunal mucosa during surgery. Our results are more in line with older culture-based studies on samples collected by needle aspiration from the jejunum of intestinally healthy individuals during abdominal surgery.

This investigation has some notable limitations. There might be differences in the microbiota of obese patients versus a normal weight population. Obesity has earlier been associated with increased risk of small intestinal bacterial overgrowth^34^ in which case the bacterial load in jejunum from non-obese might be even lower than observed in this study. On the other hand, our patients were subjected to a 3 week low calorie diet with an unknown impact on the jejunal microbiota. Further, the patients were in a fasting state. Earlier publications have found the small intestine to harbor more bacteria after meals^35^ and that environmental and food-related bacteria then make up a considerable part of the findings^36^. Therefore, as the goal of this study was to describe a normal physiological core microbiota, data from a fasting state might be preferable.

The antibiotic prophylaxis in this study, trimethoprim/sulfamethoxazole (TMP/SMX), is active against both gram negative and gram positive bacteria, but less effective against anaerobic bacteria. The antibiotic prophylaxis could interfere with results from cultivation. However, in 1978, Corrodi et al.^25^ collected jejunal content by sterile needle aspiration in eight obese patients during a gastric bypass procedure without antibiotic prophylaxis. They found 63% of samples to be cultivable sterile, even more than in our material (51.7%). The 16S deep sequencing is less likely to be noticeably affected by the antibiotics given only 2 h before surgery. Finally, although the surgeons were instructed to rub the sample collection swab firmly against the intestinal mucosa it could be that some mucosa-associated bacteria were not effectively sampled.

The strengths of this study are a high number of subjects compared to previous studies, the investigation of two separate jejunal segments from each patient and the use of surgically collected mucosal samples free of contamination from other parts of the gastrointestinal tract. Future studies on the small intestine should attempt to reduce the sampling biases by also using surgically collected samples. Unfortunately, it is a significant challenge to obtain these samples from patients not a priori in need of surgery on the small intestines. Sterile needle sampling during other types of elective abdominal surgery as used in the older culture-based studies, could represent an alternative. Although ethical and patient safety aspects need to be re-evaluated by contemporary experts in both ethics and clinical medicine, this might represent an acceptable approach in order to provide reliable data from the understudied segment of the gastrointestinal tract most essential for nutrient uptake and probably also host-microbe interactions.

## Conclusion

Proper sample collection methods is crucial for studies on the small intestine. To the best of our knowledge, this is the largest study of the jejunal bacterial microbiota collected surgically on intestinally healthy patients. Our data fail to demonstrate a jejunal resident core microbiota. Most species identified by both cultivation and deep sequencing appear only sporadically with high intra-individual differences and also considerable differences between the upper and mid segments in each patients.

## Patients and methods

### Population

Sixty patients scheduled for gastric bypass surgery at Vestfold Hospital Trust (SiV HF) were consecutively enrolled from December 2017 to September 2018. The study was approved by The Regional Committee for Medical and Health Research Ethics in the South-Eastern Norway Regional Health Region (2017/106 REK sør-øst D). All methods were performed in accordance with this approval and in accordance with the relevant guidelines and regulations. Informed consent was obtained from all participants. There were no predefined exclusion criteria.

All patients were prescribed a preoperative low-calorie diet (< 1000 kcal/day) 3 weeks before surgery, and recommended a preoperative weight loss of approximately 5%. Patients underwent fasting for solid foods a minimum of 6 h before surgery, and fluids were withheld 2 h before the procedure. Standard per oral preoperative antibiotic prophylaxis was given in the form of TMP/SMX (160 mg/800 mg) 2 h prior to surgery. All patients were examined preoperatively by a surgeon and there were no evidence of intestinal disease.

### Surgical sample collection

A standard laparoscopic gastric bypass was performed in all patients with an antegastric antecolic Roux-en-Y configuration using linear staplers. Four microbiological samples were collected from the openings of the small bowel prior to forming the two intracorporal anastomoses. Two samples were taken 60 cm from the ligament of Treitz prior the gastrojejunostomy, and two 120 cm further along the jejunum (180 cm from the ligament of Treitz) before creating the jejunojejunostomy. The swab from a standard Transwab medium (MWE, Medical Wire, UK), was introduced through a clean laparoscopy trocar and rubbed against the luminal wall to absorb the jejunum mucosal secretion. One sample from each site was cultivated within 2 h. The other pair of samples were frozen at − 70° for later DNA extraction.

### Sample cultivation and identification of bacterial colonies

50 μl of vortexed content from the Transwab medium was distributed on blood, chocolate, MacConckey and Sabouraud Dextrose agars respectively for incubation in 5% CO_2_ enriched air at 37 °C for 5 days. The same amount was spread on blood and Menadione agar plates and inoculated to a Thio broth and incubated anaerobically for 5 days. Growth was evaluated by experienced lab technicians and all colony variants were submitted for matrix assisted laser desorption/ionization-time of flight mass spectrometry (MALDI-TOF) (Bruker Daltonics, Bremen, Germany) identification using the Biotyper version 4.1.70.0–4.1.90.0 software. Scores above 2000 with consistent naming (category A) were accepted for identification at the species level. Scores between 1700 and 1999 were accepted for a genus level identification. For each species, colonies were quantified on the plate with the most abundant growth.

### Quantification by microbial culture

The swab in the Transwab kit absorbs 150 μl of sample material. The Transwab tube contains 1050 μl of solution, giving a 1:8 dilution of the sample. Provided 50 μl are spread on an agar plate, one colony (or growth in broth exclusively) corresponds to 1 × 8 × 20 = 160 cfu/ml in the original sample which was our lower limit of detection. Quantification by bacterial growth was performed according to Table 4.

**Table 4:** Quantification by microbial culture.

Growth	Colonies on plate	Cfu/ ml	Growth
No growth (lower limit of detection)	0	< 160	Non-substantial < 1600 cfu/ml
Single colonies/ broth only	1–9	160– < 1600	
Sparsely growth	10–49	1600– < 8000	Substantial ≥ 1600 cfu/ml
Moderate growth	50–100	0.8–1.6 × 10^4^	
Abundant growth (upper limit of detection)	> 100	> 1.6 × 10^4^	

### Quantification by real-time PCR

The quantitative 16S rRNA gene-PCR was based on the dual priming oligonucleotide (DPO) principle to avoid interference from human DNA^37^. The 5-end of the primers were modified according to Dyrhovden et. al.^38^ (16S_DPO_Short-F: 5’-AGAGTTTGATCMTGGCTCAIIIIIAACGCT-3’ and 16S_DPO_Short-R: 5’-CGGCTGCTGGCAIIIAITTRGC-3’). The universal anti-sense probe was designed for this study and placed in a highly conserved region of the 16S rRNA gene (Escherichia coli 16S rRNA position 360 to 341)^39^ (16S-Pb: FAM-CCYACTGCTGCCTCCCGTAG-BBQ). The PCR reaction mixture consisted of 12.5 µL Premix Ex Taq Mastermix (TaKaRa Bio, Kusatsu, Japan), 1.5 µL of each primer (from a 10 µM solution), 0.5 µL probe (from a 10 µM solution), 7 µL PCR grade water and 2 µL of template giving a total reaction volume of 25 µL. The PCR was run on a QuantStudio5 real-time PCR instrument (ThermoFisher) using a two-step thermal profile: (1) Enzyme activation at 95 °C for 30 s (2) melting at 95 °C for 10 s (3) annealing/extension at 60 °C for 20 s. Step (2) and (3) were repeated 40 times.

*Streptococcus pneumoniae* was selected as a quantitative standard due to its similarity to the other bacteria in the mitis group constituting an important part of the small intestinal microbiota. It also possesses four copies of the 16S rRNA gene which is close the estimated average number of 16S copies in bacterial genomes^40,41^. We extracted total nucleic acids from a heavy suspension of *Streptococcus pneumoniae* ATCC 49619 in PCR grade water using a MagNaPure Compact automated extractor (Roche, Mannheim, Germany). The DNA concentration in two individual samplings of the eluate was measured on a Qubit Fluorometer (Qiagen). Based on these measurements (37.3 and 39.3 ng/µl; average 38.3 ng/µl) and a genome size of 2,096,423 basepairs (ATCC 49619/GenBank accession GCA_003966485.1) we calculated the concentration of *S. pneumoniae* in our eluate to be 1.69 × 10^7^ genomes/µl. From this eluate we made a ten-fold dilution series from 1.69 × 10^7^ to 1.69 × 10^–2^. Each of the nine dilution steps was run in triplet in the quantitative real-time 16S PCR. The PCR was found to be linear down to dilution step 7, i.e. 1.69 × 10^0^ genomes/µl with an average Ct-value of **34.17** (Supplementary Fig. S2). Provided 2 µl of template and 4 copies of the 16S rRNA gene per genome this corresponds to approximately 14 target copies per PCR reaction. Taking into account the 1:833 dilution of our clinical samples during sample collection and DNA extraction, it further corresponds to 2816 bacterial genomes/ml of jejunal content which was therefore our lower limit of molecular quantification.

The *S. pneumoniae* 1.69 × 10^6^ dilution step was included as a standard in the subsequent analysis of the jejunal samples. The standard was run in triplet and the average Ct-value used to adjust the quantitative estimates for the jejunal samples. The observed inter-run variation for the standard was small (average Ct-values 31.25, 31.11 and 30.93 respectively). The estimated genome copy number per PCR reaction for the standard was 2 × 1.69 × 10^1^ i.e. 33.8 genomes which corresponds to ~ 2.9 × 10^3^ bacterial genomes/ml in a jejunal sample.

### Sample preparation and DNA extraction for 16S deep sequencing

Two-hundred μl of nuclease-free water (Ambion, Thermo Fisher Scienfic) and 450 μl sample solution from the Transwab media (MWE, Medical Wire, England) were transferred to Matrix E glasses (mpbio, MP Biomedicals, United States) and run on a FastPrep 24 instrument for 2 × 45 s. After bead-beating, the samples were centrifuged for 2 min at 13,000 rpm. Thereafter 200 μl of supernatant from each sample was used for DNA extraction and purification on a QIAsymphony automated extractor using the “DSP DNA Mini kit” (Qiagen, Hilden, Germany).

### Negative controls

Unused Transwab sample collection tubes from the two batches used in this study were included as negative controls. In addition, one Transwab tube from each batch was spiked with 1 μl of a 0.5 McFarland suspension of *Legionella pneumophila* (corresponding to 1.5 × 10^5^ bacterial cells) and included as weak positive extraction controls. Each extraction set-up therefore included two negative and two weak positive controls. Five extraction set-ups were necessary to process all samples, resulting in a total of ten negative and ten weak positive controls. Air-swabs were not included as negative controls in this study.

### 16S deep sequencing

Deep sequencing of the 16S rRNA gene was based on the Illumina V3-V4 16S metagenomics protocol with some modifications as described previously^38^: PCR amplification of the V3-V4 region was done as a real time PCR reaction on a LightCycler 480 PCR instrument (Roche) using the TBGreen Premix Ex Taq (TaKaRa, Shiga, Japan) mastermix instead of the KAPA HiFi HotStart ReadyMix. The PCR mixture consisted of 12.5 μl mastermix, 8.5 μl PCR-grade water, 1 μl of each primer (from a 10 μM solution, giving a final concentration of 0.4 μM in the PCR) and 2 μl template. After an initial polymerase activation step of 30 s at 95 °C the thermal profile included 45 cycles of 20 s at 95 °C (melting), 30 s at 60 °C (annealing), and 30 s at 72 °C (extension). The PCR products from the real time TBGreen reaction were used directly in downstream steps. The rest of the procedure was performed according to the Illumina protocol without further modifications. Sequencing was done on a MiSeq instrument (Illumina, San Diego, CA) using the Miseq reagent kit V3 (2 × 300 bp reads).

### Sequence data analysis

After sequencing, FASTQ-files were analyzed using the RipSeq NGS software (Pathogenomix, Santa Cruz, CA). After merging of R1 and R2 files, sequences shorter than 300 base pairs were removed before de novo clustering into operational taxonomic units (OTUs) using a 99% similarity threshold. OTUs with fewer than 50 sequences were rejected. Remaining OTUs were annotated using a blast search against the Pathogenomix Prime database. For an unambiguous species-level identification, we required ≥ 99.0% homology with a high-quality reference sequence combined with a minimum distance of  > 0.8% to the next alternative species. For hits above 99% but with less than 0.8% distance to the next alternative species the alternative species is presented in parenthesis. Slashed results were used for OTUs that obtained identical scores against more than one species. Homologies between 97 and 99% qualified for genus-level identification.

### Elimination of chimera and contaminant background DNA

Chimeric OTUs were filtered from all samples using the RipSeq NGS chimera check. Sequencing results from all twenty negative/weak positive controls were pooled. The most abundant contaminant species (*Cutibacterium acnes, Ralstonia pickettii* and *Staphylococcus capitis/caprae/epidermidis, Aquabacterium* and *Hydrotalea flava)* were highly consistent across all controls and used to define sample-specific cutoffs for valid identifications^42^. All species/sequence-types detected in any of the negative controls were removed from the sample sequencing results unless when they appeared in higher concentrations than all the most abundant contaminants listed above. All cultured bacteria were accepted as valid findings. Some of the cultured species were also represented in the negative sequencing controls, and therefore could not be included based on the sequencing data. This illustrates the value of combining two independent detection principles. A complete list of bacteria identified in our negative/weak positive sequencing controls is provided in Supplementary Table S4. Finally, species appearing only once among all the 120 samples, and species considered clearly biologically unexpected were removed. A complete list of rejected identifications is presented in Supplementary Table S5.

## Supplementary Information

Supplementary Information 1.

Supplementary Table S2.

Supplementary Table S3.

Supplementary Table S4.

Supplementary Table S5.

## Data Availability

The data for this study have been deposited in the European Nucleotide Archive (ENA) at EMBL-EBI under accession number PRJEB46597 (https://www.ebi.ac.uk/ena/browser/view/PRJEB46597).
