# Supplementary material for: Investigating the human jejunal microbiota
Source: Sci Rep. 2022 Jan 31;12:1682. doi: 10.1038/s41598-022-05723-9 (PMC8803847; doi:10.1038/s41598-022-05723-9)
Supplement: Supplementary file 1 — Supplementary Information 1. [file 41598_2022_5723_MOESM1_ESM.docx]

**SUPPLEMENTARY INFORMATION**

**Investigating the human jejunal microbiota**

Heidi Cecilie Villmones ^1^, Marius Svanevik^2,3^, Elling Ulvestad^4,6^, Tore Stenstad^5^, Inger Lill Anthonisen^1^, Randi Monsen Nygaard^6^, Ruben Dyrhovden^6^, Øyvind Kommedal^4,6^

^1^ Department of Microbiology, Vestfold Hospital Trust, 3103 Tønsberg, Norway

^2^ Department of Gastrointestinal Surgery, Vestfold Hospital Trust, 3103 Tønsberg, Norway

^3^ Morbid Obesity Center, Vestfold Hospital Trust, 3103 Tønsberg, Norway

^4^ Department of Clinical Science, University of Bergen, 5021 Bergen, Norway

^5^ Department of Infectious Medicine, Vestfold Hospital Trust, 3103 Tønsberg Norway

^6^ Department of Microbiology, Haukeland University Hospital, 5021 Bergen, Norway

Corresponding author Heidi Cecilie Villmones, Department of Microbiology, Vestfold Hospital Trust, Postbox 2168, 3103, Tønsberg, Norway. Tel.: +47 3334598. E-mail: heivil@siv.no

**Supplementary Table S1. Cultivated bacterial species from jejunum by frequency**

| **Growth (genus)** | **Growth (species)** | **Proximal segment (*n*)** | **Mid segment (*n*)** | **Patient level (*n*)*** |
| --- | --- | --- | --- | --- |
| **Streptococcus** | *S. salivarius/vestibularis* | **13** | **6** | **15** |
|  | *S. parasanguinis* | **9** | **3** | **10** |
|  | *S. mitis/oralis* | **7** | **1** | **7** |
|  | *S. sanguinis* | 1 | 1 | 1 |
|  | *S. australis* | 1 |  | 1 |
|  | *S. sobrinus* |  | 1 | 1 |
| ***Rothia*** | *R. mucilaginosa* | 6 | 2 | 6 |
|  | *R. dentocariosa* | 1 | 1 | 1 |
| ***Actinomyces*** | *A. odontolyticus* | 4 | 3 | 5 |
|  | *A. graevenitzii* | 1 |  | 1 |
| ***Haemophilus*** | *H. parainfluenzae* | 3 | 2 | 5 |
|  | *H. haemolyticus* | 1 | 1 | 2 |
| ***Neisseria*** | *N. parahaemolyticus* | 3 |  | 3 |
|  | *N. flavescens/subflava* | 2 | 1 | 3 |
|  | *N. flava* | 1 |  | 1 |
|  | *N. elongata* |  | 1 | 1 |
| ***Micrococcus*** | *M. luteus* | 3 | 1 | 4 |
| ***Cutibacterium*** | *C. acnes* | 3 |  | 3 |
|  | *C. avidum* |  | 1 | 1 |
| ***Staphylococcus*** | *S. aureus* | 1 | 1 | 2 |
|  | *S. epidermidis* | 1 | 1 | 1 |
|  | *S. pasteurianus* | 1 |  | 1 |
|  | *S. warneri* |  | 1 | 1 |
|  | *S. hominis* |  | 1 | 1 |
|  | *S. capitis* |  | 1 | 1 |
| ***Gemella*** | *G. sanguinis* | 1 |  | 1 |
|  | *G. haemolysans* |  | 1 | 1 |
| ***Lactobacillus*** | *L. gasseri* | 1 |  | 1 |
|  | *L. fermentum* |  | 1 | 1 |
|  | *L. salivarius* |  | 1 | 1 |
| ***Aggregatibacter*** | *A. segnis* | 1 |  | 1 |
| ***Veillonella*** | *V. parvula* | 1 |  | 1 |
|  | *Veillonella sp.* |  | 1 | 1 |
| ***Klebsiella*** | *K. pneumoniae* | 1 | 1 | 1 |
| ***Enterococcus*** | *E. faecalis* |  | 1 | 1 |
| ***Corynebacterium*** | *C. tuberculostearicum* |  | 1 | 1 |
|  | *C. aurimucosum* |  | 1 | 1 |
| ***Brachybacterium*** | *Brachybacterium sp.* |  | 1 | 1 |
| ***Dietzia*** | *Dietzia sp.* |  | 1 | 1 |
| ***Ponticoccus*** | *P. gilvus* |  | 1 | 1 |
| ***Candida*** | *C. albicans* |  | 1 | 1 |

* Both segments combined

**Supplementary Table S2. List of species in jejunum alphabetical and by abundance**

Separate EXCEL-table

**Supplementary Table S3. A comparison of findings by microbial culture versus by deep sequencing**

Separate EXCEL file

**Supplementary Table S4. List of identifications from negative and positive negative controls**

Separate EXCEL file

**Supplementary Table S5. Rejected identifications not present in the controls**

Separate EXCEL file

**Supplementary Figure S1. Relative distribution of most abundant phyla in (a) proximal part of jejunum and (b) jejunal mid-segment.** Samples are sorted by increasing bacterial concentration. Samples with concentrations below the level of quantification (Ct-value ≥34.17) are sorted by name on the left side (35 proximal samples 1j-60j and 38 mid-segment samples 1i-60i)

(a)


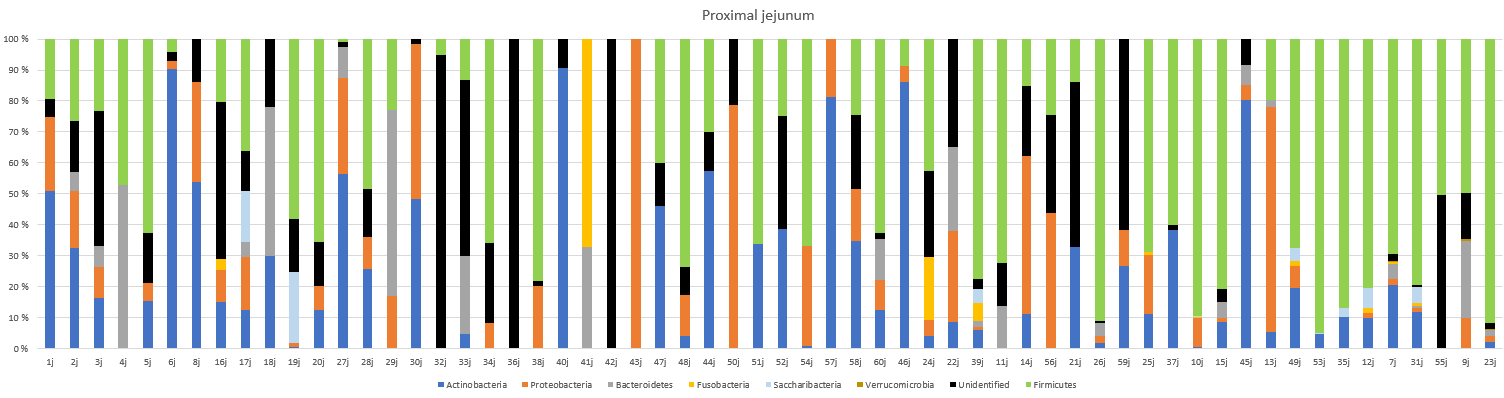


(b)


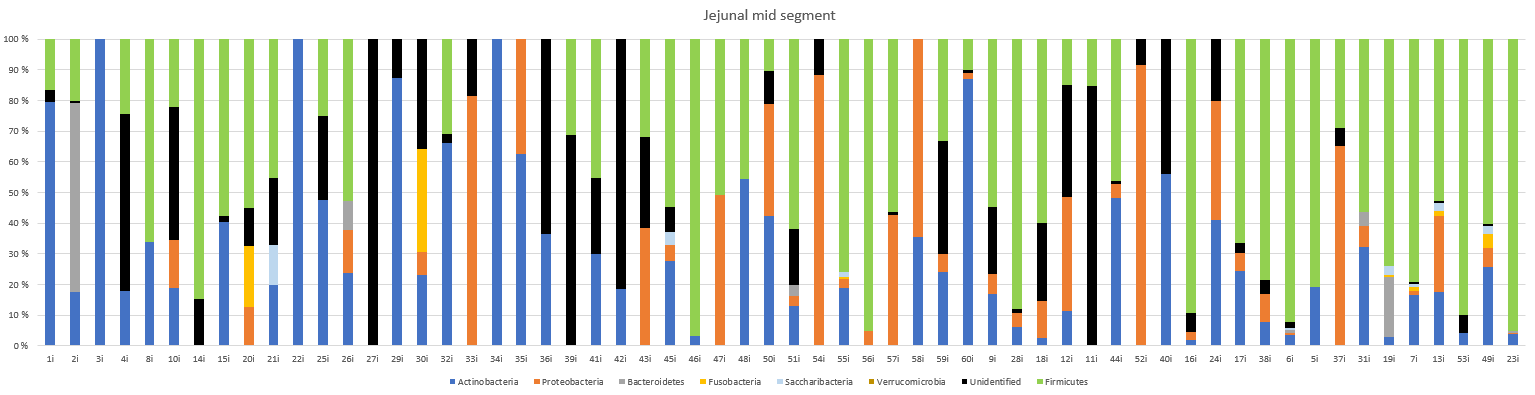


**Supplementary Figure S2. 10-fold dilution series of *S. pneumonia***


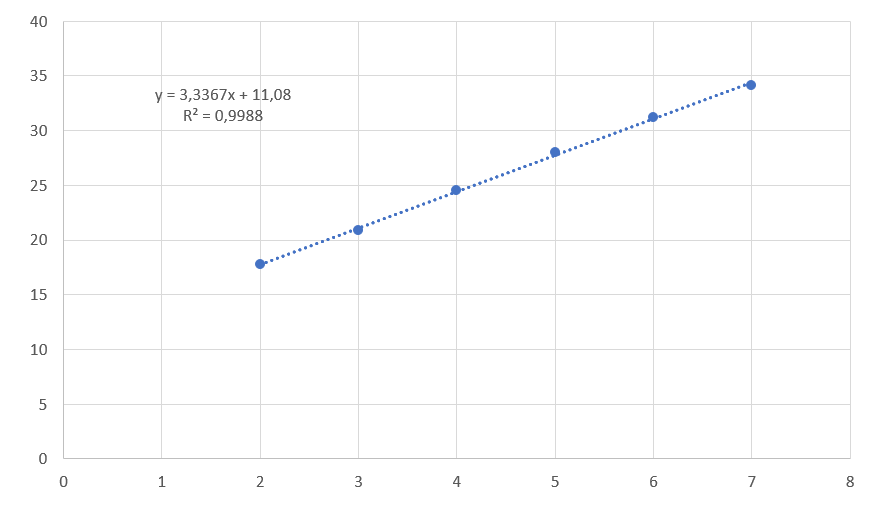
Y-axis: PCR Ct-values. X-axis: 10-fold dilutions from 10^2^ (x=2) to 10^7^ (x=7).
